# Molecular Mechanisms and Treatment Strategies for Helicobacter pylori-Induced Gastric Carcinogenesis and Mucosa-Associated Lymphoid Tissue (MALT) Lymphoma

**DOI:** 10.7759/cureus.60326

**Published:** 2024-05-15

**Authors:** Athena Myrou

**Affiliations:** 1 Department of Internal Medicine, American Hellenic Educational Progressive Association (AHEPA) University Hospital, Thessaloniki, GRC

**Keywords:** treatment strategies, molecular mechanisms, malt lymphoma, gastric cancer, helicobacter pylori

## Abstract

*Helicobacter pylori *has been classified as a class I carcinogen by WHO because of its primary involvement in the development of gastric cancer and mucosa-associated lymphoid tissue (MALT) lymphoma. This review focuses on understanding the molecular pathophysiological mechanisms that operate within intracellular transduction pathways and their relevance in the treatment strategies for the two main diseases caused by *H. pylori. H. pylori *virulence factors such as cytotoxin-associated gene A and vacuolating cytotoxin A genotypes, inflammatory mediators, *H. pylori*-induced microRNA deregulation, alterations in autophagy proteins and regulators, and changes in DNA methylation are some of the molecular mechanisms that play essential roles in *H. pylori* infection and gastric carcinogenesis. The discovery of novel treatment strategies that target the deregulated intracellular transduction pathways in gastric carcinogenesis and MALT lymphoma is critical. *H. pylori *eradication (HPE) is not limited to *H. pylori*-dependent low-grade MALT lymphoma and may be used in patients with high-grade diffuse large B-cell lymphoma (DLBCL) (de novo or DLBCL-MALT lymphoma). The loss of *H. pylori *dependency and high-grade transformation appear to be distinct events in the progression of gastric lymphoma. Interestingly, patients with *H. pylori-*positive gastric DLBCL without histological evidence of MALT lymphoma (pure gastric DLBCL) may respond to HPE therapy.

## Introduction and background

*Helicobacter pylori* is a specific type of bacteria that belongs to a separate group known as *Helicobacter* species. Over the course of the 20th century, anatomists and pathologists have noticed the presence of spiral organisms in the human gastric mucosa. However, it is believed that microorganisms cannot survive in the acidic environment of the stomach [1]. This belief was challenged by Australian pathologists Robin Warren and Barry Marshall, who isolated *H. pylori* from human gastric mucosal specimens and successfully cultured them in vitro in 1983. Their groundbreaking discovery was recognized by the Nobel Prize for Medicine and Physiology in 2005 [2].

The pathophysiology of *H. pylori* infection (*Hp-I*) is characterized by two main factors: (i) factors that promote colonization of the host, such as motility, adhesion, urease, flagella, and pATPase and (ii) factors that contribute to tissue damage due to the presence of the bacterium, including lipopolysaccharide (LPS), *H. pylori* neutrophil-activating protein (HP-NAP), cytotoxin-associated gene A (CagA), vacuolating cytotoxin A (VacA), and heat shock proteins [3].

*Hp-I* is known for its T-cell hyporesponsiveness, with the existing response primarily consisting of a T helper 1 (Th1) response that may be triggered by HP-NAP and cell wall LPS. HP-NAP has been shown to shift antigen-specific T-cell responses from a predominant Th2 phenotype to a polarized Th1 phenotype characterized by high levels of interferon and tumor necrosis factor (TNF) production [4].

*H. pylori* is a significant cause of gastritis, gastric and duodenal ulcer disease, and increases the risk of gastric cancer. It has also been implicated in the development of mucosa-associated lymphoid tissue (MALT) lymphoma, which remits after eradication of the infection. Consequently, *H. pylori* has been classified as a class I carcinogen by WHO [5,6].

The following information pertains to the two diseases linked to *Hp-I*: gastric cancer and MALT lymphoma. This is a well-known and well-described phenomenon known as *H. pylori*-induced carcinogenesis.

## Review

Gastric cancer and H. pylori

CagA-Related Pathways, H. pylori, and Gastric Cancer

The CagA pathogenicity island is a component of *H. pylori* that encodes a type 4 secretion system (T4SS) and the virulence CagA protein. This oncoprotein is delivered into gastric epithelial cells via bacterial T4SS, which modifies the phenotype of epithelial cells and perturbs multiple host signaling pathways involved in gastric carcinogenesis.

CagA-positive genotypes can have a C-terminal EPIYAU region. C-EPIYAU-positive (+) CagA, upon delivery in epithelial cells, is phosphorylated by the Src family tyrosine kinase, and the phosphorylated CagA then binds and activates SHP2. The created complex (CagA-SHP2 complex) activates the mitogen-activated protein kinase/extracellular signal-regulated kinase (MAPK/ERK) pathway [7].

In contrast, C-EPIYAU negative (-) CagA interacts with Janus activated kinase 2 (JAK2), causing phosphorylation and activation of signal transducer and activator of transcription 3 (STAT3). Both STAT3 and JAK2/MAPK pathways can also be induced by interleukin-6-glycoprotein130 (IL-6-gp130) interaction.

It has been shown that CagA can phosphorylate gp130 receptors, regardless of the status of C-EPIYA [8]. The aforementioned studies indicate that CagA has a significant impact on the phenotype of epithelial gastric cells. Specifically, CagA achieves this effect by activating the MAPK/ERK pathway, inhibiting focal adhesion kinase-mediated cell-matrix interactions, and inhibiting polarity-regulating kinase partitioning-defective 1b (PAR1b) through its CM sequence. Research has shown that PAR1b inactivates a RhoA-specific guanine nucleotide exchange factor (GEF), particularly GEF-H1, by phosphorylation, thereby suppressing cortical actin and stress fiber formation and amplifying the hummingbird phenotype [3]. Studies by Bessede et al. have demonstrated that the hummingbird phenotype is involved in gastric carcinogenesis and that hummingbird gastric cells exhibit many properties of cancer stem cells [9].

The promotion of gastric cancer development and progression by *H. pylori *through CagA is facilitated by another mechanism that involves the inactivation of p53. Upon insertion into gastric epithelial cells, CagA interacts with apoptosis-stimulating protein p53 (ASPP2), suppressing p53 apoptotic function [10]. Research conducted by Buti et al. demonstrated that *H. pylori*-infected cells treated with doxorubicin, a drug that activates p53, exhibit increased resistance to apoptosis compared to even uninfected cells [11]. IL-8 plays a crucial role in angiogenesis, adhesion, migration, and invasion of cancer cells, and its function in these processes is well-defined and established [12,13]. *H. pylori *induces IL-8 expression through two pathways: (1) the activation of the oxidant-sensitive nuclear transcriptional factor kappa B via the classic pathway and alternatively through MAPK stimulation and (2) the activation of the pathway related to transcription factor AP-1. The interaction between cytosolic nucleotide binding and oligomerization domain 1 after delivery of *H. pylori* peptidoglycan in host cells for both mechanisms is essential [14] Although Ando et al. reported that IL-8 expression is present in CagA-negative *H. pylori* genotypes [15], Peng et al. observed the upregulation of IL-8 expression in both strains [16].

MicroRNAs’ (miRNAs) Participation in H. pylori-Related Gastric Cancer

miRNAs are small non-coding RNA molecules that contain approximately 22 nucleotides. They play a role in RNA silencing and the posttranscriptional regulation of gene expression. Abnormal miRNA expression after *Hp-I *may cause an unregulated inflammatory response and immune system disruption, potentially contributing to the pathogenesis of gastric cancer [17].

A study conducted by Zhu et al. revealed that CagA is involved in gastric carcinogenesis through its association with miRNA-584 and miRNA-1290, which are upregulated in CagA-transformed cells. This overexpression results in the development of intestinal metaplasia in the gastric epithelial cells of knock-in mice [18].

In addition to CagA, miRNAs also appear to play a role in the JAK2-STAT3 pathway. Miao et al. demonstrated that miRNA-375 regulates the JAK2-STAT3 pathway by affecting the expression of B-cell CLL/lymphoma 2 (BCL-2) and TWIST1 [19]. Specifically, they observed that miR-375 was downregulated in response to *Hp-I* in gastric epithelial cell lines and that this decrease in expression could mimic the oncogenic effects of the JAK2-STAT3 pathway.

The roles of miRNAs miR-204 and miR-141 in gastric cancer have been extensively studied [20,21]. In general, miR-204 has been recognized to function as a tumor suppressor, with low expression levels associated with A poor prognosis in patients with cancer. In gastric cancer, miR-204 was found to be significantly downregulated in both normal and tumor tissues infected with *H. pylori* [21]. Overexpression of miR-204 suppresses the growth of gastric tumor cells, which in turn downregulates epithelial-mesenchymal transition (EMT) inducers, as the SOX4 transcription factor interacts with EMT transcriptional inducers, which are crucial in cancer and may contribute to therapy resistance [22].

Autophagy Pathway and H. pylori-Related Gastric Cancer

Defective autophagy can lead to the accumulation of damaging substances, inflammation, and an increased risk of cancer. *H. pylori *and its virulence factors, such as CagA and VacA, interact with the host’s autophagic system in intricate ways. This has implications for the pathophysiology of several diseases, including infections and cancer [23,24].

Castaño-Rodriguez et al. investigated the impact of *H. pylori*’s *Hp-I* component on host cell gene expression and the relationship between key genetic polymorphisms involved in autophagy and gastric cancer. Their results revealed that autophagy proteins were severely impaired in highly virulent *H. pylori *strains, including CagA- and VacA-positive genotypes [25]. Deen et al. also examined the effects of VacA on the autophagic machinery and observed distinct responses in host cells during acute and chronic inflammation [24]. While VacA promotes autophagy in the acute phase, protecting host cells from toxicity, it impairs autophagic protection during chronic inflammation by accumulating SQSTM1. SQSTM1, also known as p62, is a multifunctional signal adapter protein involved in both autophagy and apoptosis pathways in tumor cells [26]. This accumulation is indicative of deficiencies in the autophagic pathway. Prolonged exposure to VacA leads to the gradual accumulation of SQSTM1 in infected gastric epithelial cells, especially in those infected with the toxigenic s1m1 form of VacA [27]. Furthermore, these cells lack the essential lysosomal protease CTSD/cathepsin D [14], providing further evidence that chronic exposure to VacA impairs autophagy.

DNA Methylation Mechanisms, H. pylori, and Gastric Cancer

The role of DNA methylation in the regulation of gene transcription has received significant attention, particularly its involvement in carcinogenesis. Abnormal DNA methylation patterns, including hypermethylation and hypomethylation, have been associated with a wide range of human cancers. Hypermethylation typically occurs at CpG islands in the promoter region and is associated with the inactivation of tumor suppressor genes in cancer. Hypomethylation, on the other hand, has been implicated in the expression of oncogenes. *Hp-I *has been shown to increase DNA methylation in gene control regions, which in turn increases the risk of gastric cancer through two pathways: chronic inflammation and aberrant promoter methylation of specific tumor suppressor genes, or oncogenes.

Chronic inflammation has been found to result in increased expression of several genes, including IL-8, nitric oxide (NO) synthase 2, IL-1β, and TNF, in diseases such as hepatitis and ulcerative colitis. These genes are commonly associated with the induction of abnormal DNA methylation during chronic inflammation [28]. The roles of IL-1β and NO are also specific. A meta-analysis of 20,000 subjects showed a positive relationship between IL-1β expression and gastric cancer, and this relationship was further strengthened by *Hp-I* [29]. IL-1β induces several effects, including the expression of NO/inducible NO synthase and E-cadherin and promoter methylation of the anti-inflammatory cytokine transforming growth factor-1 in gastric epithelial cells. NO plays a role in mediating *H. pylori*-induced DNA overmethylation of tumor suppressor genes [30].

*H. pylori *strains suppress the expression of the p53 tumor suppressor gene through hypermethylation or deletion of the ARF tumor suppressor gene (p14ARF). Another tumor suppressor gene that appears to be degraded by *H. pylori *through hypermethylation is FOXD3, which plays a crucial role in early embryonic development. Additionally, *H. pylori *participates in miRNA methylation. For example, Kiga et al. reported that DNA methylation of the miR-210 gene is increased in* H. pylori*-positive human gastric biopsies compared to those in *H. pylori*-negative controls [31]. MiR-210 is associated with hypoxia and overexpressed in tumors.

Other Signaling Pathways, H. pylori, and Gastric Cancer

According to previous reports, *Hp-I* has been found to increase the phosphorylation levels of Raf kinase inhibitor protein (pRKIP) in gastric adenocarcinoma (AGS) cells both in vitro and in vivo. It has been proposed that *H. pylori*-induced pro-survival signaling in gastric epithelial cells leads to the activation of RKIP through a feedback response. Increased expression of pro-tumorigenic proteins, such as the epidermal growth factor (EGF) receptor and MAPKs, enhances this interaction [32].

**Figure 1:**
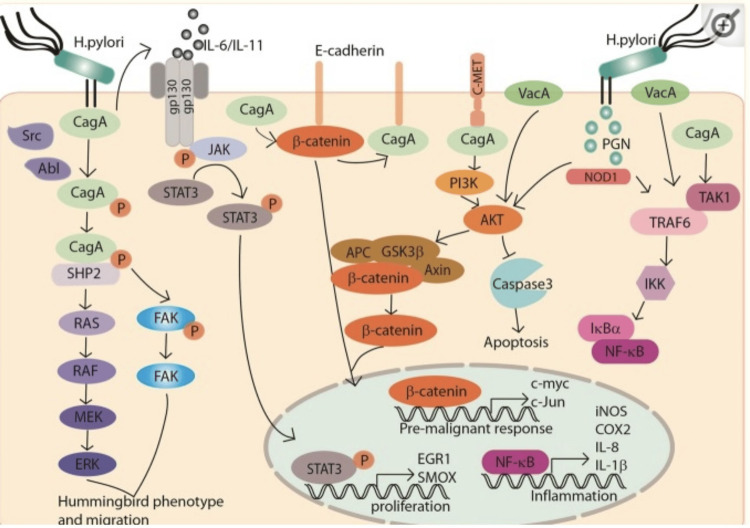
Molecular pathogenesis of H. pylori in gastric carcinogenesis

The role of STAT3, which connects *Hp-I* with gastric oncogenesis, is crucial. *H. pylori *triggers STAT3 signaling and induces STAT3-dependent cyclooxygenase-2 (COX-2) expression both in vitro and in vivo. Immunohistochemical staining revealed that *H. pylori*-positive gastritis tissues exhibited markedly higher levels of pSTAT3(Tyr705) than did *H. pylori*-negative tissues. High pSTAT3(Tyr705) levels are correlated with intestinal metaplasia and dysplasia, suggesting that pSTAT3(Tyr705) may be useful for the early detection of gastric tumorigenesis. Additionally, a strong positive correlation between STAT3/pSTAT3(Tyr705) levels and COX-2 expression has been identified in gastritis and gastric cancer tissues [33]. Moreover, through the activation of STAT3,* Hp-I *deregulates multiple tumorigenic genes, which may contribute to the initiation and progression of gastric cancer [34] (Figure 1).

Germ-line-encoded receptors, referred to as pattern recognition receptors, are essential to produce mature pro-inflammatory cytokines that are vital for both Th1 and Th2 responses. Current evidence indicates that Toll-like receptors, along with nucleotide-binding oligomerization domain-like receptors and related polymorphisms in their corresponding genes, are involved in both the recognition of* H. pylori *and gastric carcinogenesis [35].

The relationship between inflammation and cancer is well established, with chronic inflammation being a significant risk factor for cancer development. Individuals infected with *H. pylori *are at a higher risk of developing chronic inflammation due to the bacteria’s use of virulence factors such as CagA, VacA, and peptidoglycan to upregulate proinflammatory cytokines, including IL-1, IL-6, IL-8, TNF-α, and NF-κB in gastric epithelial cells and circulating immune cells [36]. This production of cytokines triggers the activation and migration of leukocytes and regulates a cascade of cytokines, chemokines, and adhesions. Granulocyte-macrophage colony-stimulating factor, a growth factor that facilitates white cell differentiation, has been detected in *H. pylori*-infected antral biopsies and human gastric epithelial cells [37]. Additionally, the expression of the inflammatory modulator cyclooxygenase-2, which converts arachidonic acid to prostaglandins to induce inflammatory reactions, is significantly higher in* H. pylori*-infected gastric epithelial cells [38].

H. pylori, Molecular Transduction Pathways, and Gastric Cancer: Treatment Applications

The urgent need for innovative therapeutic approaches that delve into the intricate mysteries and mechanisms underpinning gastric carcinogenesis at the molecular level is crucial. This should focus on intracellular signaling pathways that are disrupted in the disease. miRNAs are pivotal in regulating development, differentiation, immunity, and carcinogenesis.

Miao et al. discovered that miR-375 was downregulated in response to *Hp-I* in gastric epithelial cell lines, which mimicked the oncogenic effects of the JAK2-STAT3 pathway. Furthermore, pretreatment with small interfering RNAs targeting JAK2 prevents the proliferation and migration of gastric epithelial cells in response to *Hp-I*, offering a potential therapeutic target [19]. In another study, it was demonstrated that miR-141 expression was decreased in *H. pylori*-positive tissues and that this low expression correlated with a higher invasion ability of gastric cancer cells through the STAT4 pathway. Therefore, targeting the STAT4 pathway and increasing miR-141 expression may be beneficial in patients with gastric cancer [39]. Additionally, miR-101 has been reported to function as a growth-suppressive miRNA in *H. pylori*-related gastric carcinogenesis, with its suppressive effects mediated mainly by repressing suppressor of cytokine signaling 2 expression. These findings suggest novel therapeutic approaches for these targets [40].

Overexpression of Gastrokine 1 may suppress the carcinogenic effects of CagA, which is a potential target for inhibiting* H. pylori* [41]. Deleting peptidyl-prolyl cis-trans isomerase (PPIase) prevents *H. pylori* from stimulating human gastric epithelial cell (AGS) proliferation. Ectopic expression of *H. pylori *PPIase promotes AGS cell proliferation and anchorage-independent growth. Researchers have studied the biochemical mechanisms of PPIase-induced effects on early signaling events in the mitogenic signaling pathways. *H. pylori* PPIase increased basal and EGF-stimulated phosphorylation of ERK and EGF receptors at Tyr1086. Treatment with mitogen-activated protein/extracellular kinase inhibitors completely blocked PPIase-induced cell proliferation. Therefore, *H. pylori* PPIase may be a promising target for therapeutic interventions in patients with gastric cancer [42].

Gastric cancer therapy uses various antigens, including carbohydrate type 2 blood group antigens. Studies have shown that the correlative sources of the specific gastric biomarkers, COX-2 and LeY, are involved in the MAPK pathway [43]. The anti-LeY antibody downregulated COX-2 expression via the MAPK pathway, which could aid in gastric cancer treatment. SOX2 controls important signaling pathways, regulates expression, and provides new therapeutic options for a subset of gastric cancers with SOX2 dysregulation [44].

MALT Lymphoma, Diffuse Large B-Cell Lymphoma (DLBCL), and H. pylori

Gastric MALT lymphoma is a low-grade B-cell non-Hodgkin lymphoma. Most cases (approximately 90%) were directly related to *Hp-I*, thus eliminating the bacterium as the primary treatment option [45].

Changes in miRNA expression, such as overexpression or downregulation of specific miRNA strands (e.g., miR-150, 550, 124a, 518b, 539, and 203), have been associated with gastric lymphagenesis, whereas underexpression of miR-34a is linked to the transition from MALT to DLBCL lymphoma. Additionally, high DNA methylation levels have been correlated with *Hp-I* [46].

Hamoudi et al. conducted a study that established a strong link between abnormal activation of the NF-κB signaling pathway and the t (11;18) (q21; q21), t (1;14) (p22; q32), and t (14;18) (q32; q21) translocations [47]. Zhang et al. further elucidated the multistep mechanism behind this connection, explaining how these translocations produce fusion proteins that share the same NF-κB signaling pathway and directly participate in the development of cancer [48]. However, it is believed that these genetic alterations alone are insufficient for the development of lymphomas. Interactions with existing immunological stimulation are thought to be necessary for malignancy to occur [49].

A study by Hajder et al. should also be considered. Researchers performed biopsies of GML samples and analyzed the expression of BCL10 and NF-κB (p65 subunit). More than 50% of the samples displayed cytoplasmic expression of both markers, but no nuclear expression was detected. Despite this, the study did not find a correlation between the co-expression of these two markers and GML; therefore, it was concluded that the suppression of apoptosis was not a major factor in oncogenesis [50].

Although there are currently no medications available that target the NF-κB signaling pathway, this is an area of ongoing research.

Matsui et al. conducted a controlled study using C57BL/6J mice infected with *Helicobacter suis* TKY (a strain of bacteria strongly associated with GML in mice) and *H. pylori SS1* to determine whether various strains of *Lactobacillus* offered any protection against these two strains of *H. pylori*. The researchers tested five different strains of *Lactobacillus* at a concentration of 108-109 colony-forming units, administered two days and two weeks before intragastric infection with *Helicobacter*, and continued supplementation for 12 months after infection. At the end of the 12-month period, only the *Lactobacillus gasseri* SBT2055 strain protected against the formation of round protrusive lesions in the gastric fundus and suppressed the formation of lymphoid follicles in the gastric mucus layers at three months postinfection [51]. However, it remains to be seen whether this protective effect also applies to humans.

The progression of MALT lymphomas to DLCBL is commonly associated with *Hp-I*; however, data regarding pure gastric DLCBL are limited. To address this gap, Huang et al. conducted a study of 102 cases of pure gastric DLCBL. The results showed that the presence of *Hp-I* in these cases was correlated with earlier-stage lymphomas (I and II) and a higher chance of remission when compared to *H. pylori*-negative DLCBL (73% vs. 52% and 75% vs. 43%, respectively). This protective effect of *Hp-I* was attributed to the inhibition of ZEB1 by increased levels of miR-200 and higher levels of BCL6, known predictors of favorable outcomes in DLBCL cases. Therefore, pure DLBCL coexisting with *Hp-I *is less aggressive and has a better prognosis [52].

Schaberg et al. discovered a significant number of *H. pylori*-negative patients with MALT lymphoma (lacking a DLBCL component) despite the well-established connection between *Hp-I* and gastric MALT lymphomas. This finding prompted them to investigate the potential causes of the unusually low prevalence of *H. pylori* in these patients. The researchers found that treatment with antisecretory medication led to a decrease in the detection of *Hp-I* by histological examination. Therefore, it is recommended that other methods, such as serological screening, be employed to avoid false negatives when detecting *Hp-I* [53].

Advances have been made in evaluating the severity and prognosis of gastric DLBCL. Research has found that overexpression of mitotic arrest deficiency protein 2 (Mad2) is associated with cell proliferation and genetic instability, which can contribute to oncogenesis. Additionally, overexpression of Mad2 has been linked to reduced disease-free survival but not overall survival, suggesting its potential as a diagnostic criterion [54].

Furthermore, studies have reported that MLL2 protein is overexpressed in patients with gastric DLBCL and is correlated with the clinical stage of the condition. Higher levels of MLL2 are also associated with lower patient survival. However, these relationships were observed only in patients aged >60 years, limiting the scope of this prognostic tool [55].

One possible prognostic indicator is the co-expression of MYC and BCL-2 proteins, which has been shown to be associated with the severity of gastrointestinal DLBCL, patient response to chemotherapy, the likelihood of complete remission, and overall survival compared to patients who express only one of these proteins [56].

According to Kuo et al. [57], half of the patients with GML have detectable levels of CagA protein in malignant B cells. Notably, patients who had both GML and were infected with a CagA-positive strain showed a significant improvement in response to eradication compared to those infected with a CagA-negative strain [58].

Indeed, it is essential to conduct additional research to fine-tune the current and evolving diagnostic criteria and determine the optimal therapeutic approach for individuals with *H. pylori*-positive DLCBL. Given that a sizable proportion of patients exhibit favorable outcomes following antibiotic treatment alone, it is prudent to restrict the use of chemotherapy and radiotherapy to situations in which these interventions are ineffective. This observation appears to be applicable to MALT lymphoma [59].

## Conclusions

*H. pylori* is a class I carcinogen known to cause gastric cancer and MALT lymphoma. The molecular pathophysiological mechanisms underlying the development of these diseases involve various factors, such as virulence factors, inflammatory mediators, miRNA deregulation, autophagy protein alterations, and DNA methylation changes. It is essential to target these deregulated intracellular signaling pathways to develop effective novel treatment strategies for gastric carcinogenesis and MALT lymphoma. Eradication of *Hp-I* should be considered in patients with high-grade DLBCL. Additionally, it appears that the loss of *H. pylori* dependency and high-grade transformation are distinct events in the progression of gastric lymphoma.

## References

[1] Palmer ED (1954). Investigation of the gastric mucosa spirochetes of the human. Gastroenterology.

[2] Warren JR, Marshall BJ (1983). Unidentified curved bacilli on gastric epithelium in active chronic gastritis. Lancet.

[3] Freire de Melo F, Marques HS, Rocha Pinheiro SL (2022). Influence of Helicobacter pylori oncoprotein CagA in gastric cancer: a critical-reflective analysis. World J Clin Oncol.

[4] Sitkin S, Lazebnik L, Avalueva E, Kononova S, Vakhitov T (2022). Gastrointestinal microbiome and Helicobacter pylori: eradicate, leave it as it is, or take a personalized benefit-risk approach?. World J Gastroenterol.

[5] Alipour M (2021). Molecular mechanism of Helicobacter pylori-induced gastric cancer. J Gastrointest Cancer.

[6] Papagiannakis P, Michalopoulos C, Papalexi F, Dalampoura D, Diamantidis MD (2013). The role of Helicobacter pylori infection in hematological disorders. Eur J Intern Med.

[7] Backert S, Tegtmeyer N, Fischer W (2015). Composition, structure and function of the Helicobacter pylori cag pathogenicity island encoded type IV secretion system. Future Microbiol.

[8] Glowinski F, Holland C, Thiede B, Jungblut PR, Meyer TF (2014). Analysis of T4SS-induced signaling by H. pylori using quantitative phosphoproteomics. Front Microbiol.

[9] Bessède E, Staedel C, Acuña Amador LA (2014). Helicobacter pylori generates cells with cancer stem cell properties via epithelial-mesenchymal transition-like changes. Oncogene.

[10] Junaid M, Shah M, Khan A (2019). Structural-dynamic insights into the H. pylori cytotoxin-associated gene A (CagA) and its abrogation to interact with the tumor suppressor protein ASPP2 using decoy peptides. J Biomol Struct Dyn.

[11] Buti L, Spooner E, Van der Veen AG, Rappuoli R, Covacci A, Ploegh HL (2011). Helicobacter pylori cytotoxin-associated gene A (CagA) subverts the apoptosis-stimulating protein of p53 (ASPP2) tumor suppressor pathway of the host. Proc Natl Acad Sci U S A.

[12] Zarogoulidis P, Katsikogianni F, Tsiouda T, Sakkas A, Katsikogiannis N, Zarogoulidis K (2014). Interleukin-8 and interleukin-17 for cancer. Cancer Invest.

[13] Kuai WX, Wang Q, Yang XZ, Zhao Y, Yu R, Tang XJ (2012). Interleukin-8 associates with adhesion, migration, invasion and chemosensitivity of human gastric cancer cells. World J Gastroenterol.

[14] Junaid M, Li CD, Shah M, Khan A, Guo H, Wei DQ (2019). Extraction of molecular features for the drug discovery targeting protein-protein interaction of Helicobacter pylori CagA and tumor suppressor protein ASSP2. Proteins.

[15] Ando T, Peek RM Jr, Lee YC (2002). Host cell responses to genotypically similar Helicobacter pylori isolates from United States and Japan. Clin Diagn Lab Immunol.

[16] Peng YC, Ho SP, Shyu CL, Chang CS, Huang LR (2014). Clarithromycin modulates Helicobacter pylori-induced activation of nuclear factor-κB through classical and alternative pathways in gastric epithelial cells. Clin Exp Med.

[17] Chang H, Kim N, Park JH (2015). Different microRNA expression levels in gastric cancer depending on Helicobacter pylori infection. Gut Liver.

[18] Zhu Y, Jiang Q, Lou X (2012). MicroRNAs up-regulated by CagA of Helicobacter pylori induce intestinal metaplasia of gastric epithelial cells. PLoS ONE.

[19] Miao L, Liu K, Xie M, Xing Y, Xi T (2014). miR-375 inhibits Helicobacter pylori-induced gastric carcinogenesis by blocking JAK2-STAT3 signaling. Cancer Immunol Immunother.

[20] Ansari S, Yamaoka Y (2022). Helicobacter pylori infection, its laboratory diagnosis, and antimicrobial resistance: a perspective of clinical relevance. Clin Microbiol Rev.

[21] Zhou X, Li L, Su J, Zhang G (2014). Decreased miR-204 in H. pylori-associated gastric cancer promotes cancer cell proliferation and invasion by targeting SOX4. PLoS ONE.

[22] Khatoon J, Prasad KN, Prakash Rai R, Ghoshal UC, Krishnani N (2017). Association of heterogenicity of Helicobacter pylori cag pathogenicity island with peptic ulcer diseases and gastric cancer. Br J Biomed Sci.

[23] Zheng SY, Zhu L, Wu LY (2023). Helicobacter pylori-positive chronic atrophic gastritis and cellular senescence. Helicobacter.

[24] Deen NS, Huang SJ, Gong L, Kwok T, Devenish RJ (2013). The impact of autophagic processes on the intracellular fate of Helicobacter pylori: more tricks from an enigmatic pathogen?. Autophagy.

[25] Castaño-Rodríguez N, Kaakoush NO, Goh KL, Fock KM, Mitchell HM (2015). Autophagy in Helicobacter pylori infection and related gastric cancer. Helicobacter.

[26] Zhang YB, Gong JL, Xing TY, Zheng SP, Ding W (2013). Autophagy protein p62/SQSTM1 is involved in HAMLET-induced cell death by modulating apotosis in U87MG cells. Cell Death Dis.

[27] Raju D, Hussey S, Ang M (2012). Vacuolating cytotoxin and variants in Atg16L1 that disrupt autophagy promote Helicobacter pylori infection in humans. Gastroenterology.

[28] Cai Q, Shi P, Yuan Y (2021). Inflammation-associated senescence promotes Helicobacter pylori-induced atrophic gastritis. Cell Mol Gastroenterol Hepatol.

[29] Park MJ, Hyun MH, Yang JP, Yoon JM, Park S (2015). Effects of the interleukin-1β-511 C/T gene polymorphism on the risk of gastric cancer in the context of the relationship between race and H. pylori infection: a meta-analysis of 20,000 subjects. Mol Biol Rep.

[30] Lamb A, Chen LF (2013). Role of the Helicobacter pylori-induced inflammatory response in the development of gastric cancer. J Cell Biochem.

[31] Kiga K, Mimuro H, Suzuki M (2014). Epigenetic silencing of miR-210 increases the proliferation of gastric epithelium during chronic Helicobacter pylori infection. Nat Commun.

[32] Nisimova L, Wen S, Cross-Knorr S, Rogers AB, Moss SF, Chatterjee D (2014). Role of Raf kinase inhibitor protein in Helicobacter pylori-mediated signaling in gastric cancer. Crit Rev Oncog.

[33] Zhang J, Wei J, Wang Z (2020). Transcriptome hallmarks in Helicobacter pylori infection influence gastric cancer and MALT lymphoma. Epigenomics.

[34] Zhao J, Dong Y, Kang W (2014). Helicobacter pylori-induced STAT3 activation and signalling network in gastric cancer. Oncoscience.

[35] Castaño-Rodríguez N, Kaakoush NO, Mitchell HM (2014). Pattern-recognition receptors and gastric cancer. Front Immunol.

[36] Dixon BR, Hossain R, Patel RV, Algood HM (2019). Th17 cells in Helicobacter pylori infection: a dichotomy of help and harm. Infect Immun.

[37] Tang B, Li N, Gu J (2012). Compromised autophagy by MIR30B benefits the intracellular survival of Helicobacter pylori. Autophagy.

[38] He Y, Zhao X, Subahan NR, Fan L, Gao J, Chen H (2014). The prognostic value of autophagy-related markers beclin-1 and microtubule-associated protein light chain 3B in cancers: a systematic review and meta-analysis. Tumour Biol.

[39] Zhou X, Xia Y, Su J, Zhang G (2014). Down-regulation of miR-141 induced by helicobacter pylori promotes the invasion of gastric cancer by targeting STAT4. Cell Physiol Biochem.

[40] Zhou X, Xia Y, Li L, Zhang G (2015). MiR-101 inhibits cell growth and tumorigenesis of Helicobacter pylori related gastric cancer by repression of SOCS2. Cancer Biol Ther.

[41] Yoon JH, Seo HS, Choi SS (2014). Gastrokine 1 inhibits the carcinogenic potentials of Helicobacter pylori CagA. Carcinogenesis.

[42] Zhu Y, Chen M, Gong Y (2015). Helicobacter pylori FKBP-type PPIase promotes gastric epithelial cell proliferation and anchorage-independent growth through activation of ERK-mediated mitogenic signaling pathway. FEMS Microbiol Lett.

[43] Aziz F, Qiu Y (2014). The role of anti-LeY antibody in the downregulation of MAPKs/COX-2 pathway in gastric cancer. Curr Drug Targets.

[44] Hütz K, Mejías-Luque R, Farsakova K (2014). The stem cell factor SOX2 regulates the tumorigenic potential in human gastric cancer cells. Carcinogenesis.

[45] Asano N, Iijima K, Koike T, Imatani A, Shimosegawa T (2015). Helicobacter pylori-negative gastric mucosa-associated lymphoid tissue lymphomas: a review. World J Gastroenterol.

[46] Vasilatou D, Sioulas AD, Pappa V, Papanikolaou IS, Triantafyllou K, Dimitriadis GD, Papageorgiou SG (2016). The role of miRNAs and epigenetic mechanisms in primary gastric mucosa-associated lymphoid tissue lymphoma. Future Oncol.

[47] Hamoudi RA, Appert A, Ye H (2010). Differential expression of NF-κB target genes in MALT lymphoma with and without chromosome translocation: insights into molecular mechanism. Leukemia.

[48] Zhang Y, Wei Z, Li J, Liu P (2015). Molecular pathogenesis of lymphomas of mucosa-associated lymphoid tissue—from (auto)antigen driven selection to the activation of NF-κB signaling. Sci China Life Sci.

[49] Du MQ (2011). MALT lymphoma: many roads lead to nuclear factor-κb activation. Histopathology.

[50] Hajder J, Marisavljević D, Stanisavljević N, Mihaljević B, Kovcin V, Marković O, Zivković R (2014). BCL10 aberations and NF-kappa B activation involving p65 are absent or rare in primary gastric MALT lymphoma. Vojnosanit Pregl.

[51] Matsui H, Takahashi T, Øverby A (2015). Mouse models for assessing the protective efficacy of Lactobacillus gasseri SBT2055 against helicobacter suis infection associated with the development of gastric mucosa-associated lymphoid tissue lymphoma. Helicobacter.

[52] Huang WT, Kuo SH, Cheng AL, Lin CW (2014). Inhibition of ZEB1 by miR-200 characterizes Helicobacter pylori-positive gastric diffuse large B-cell lymphoma with a less aggressive behavior. Mod Pathol.

[53] Schaberg KB, Evans MF, Wilcox R, Lewis MR (2015). Antisecretory medication is associated with decreased Helicobacter pylori detection in gastric marginal zone lymphoma. Ann Diagn Pathol.

[54] Chen F, Liu S, Zhou Y, Shen H, Zuo X (2016). Mad2 overexpression is associated with high cell proliferation and reduced disease-free survival in primary gastrointestinal diffuse large B-cell lymphoma. Hematology.

[55] Ye H, Lu L, Ge B (2015). MLL2 protein is a prognostic marker for gastrointestinal diffuse large B-cell lymphoma. Int J Clin Exp Pathol.

[56] Xia B, Zhang L, Guo SQ (2015). Coexpression of MYC and BCL-2 predicts prognosis in primary gastrointestinal diffuse large B-cell lymphoma. World J Gastroenterol.

[57] Kuo SH, Yeh KH, Chen LT (2014). Helicobacter pylori-related diffuse large B-cell lymphoma of the stomach: a distinct entity with lower aggressiveness and higher chemosensitivity. Blood Cancer J.

[58] Kuo SH, Yeh KH, Wu MS (2012). Helicobacter pylori eradication therapy is effective in the treatment of early-stage H pylori-positive gastric diffuse large B-cell lymphomas. Blood.

[59] Cuccurullo R, Govi S, Ferreri AJ (2014). De-escalating therapy in gastric aggressive lymphoma. World J Gastroenterol.

